# Topical Administration of Melatonin-Loaded Extracellular Vesicle-Mimetic Nanovesicles Improves 2,4-Dinitrofluorobenzene-Induced Atopic Dermatitis

**DOI:** 10.3390/biom11101450

**Published:** 2021-10-02

**Authors:** Yoon Seon Kim, Gyeongyun Go, Chul-Won Yun, Ji-Hye Yea, Sungtae Yoon, Su-Yeon Han, Gaeun Lee, Mi-Young Lee, Sang Hun Lee

**Affiliations:** 1 Department of Medical Science, Soonchunhyang University, Asan-si 31538, Korea; agust2810@kakao.com; 2 Department of Biochemistry, College of Medicine, Soonchunhyang University, Cheonan 31151, Korea; ggy0227@naver.com (G.G.); yeajihye93@gmail.com (J.-H.Y.); lun4905@naver.com (G.L.); 3 Department of Biochemistry, BK21FOUR Project2, College of Medicine, Soonchunhyang University, Cheonan 31151, Korea; 4 Medical Science Research Institute, Soonchunhyang University Seoul Hospital, Seoul 04401, Korea; skydbs113@naver.com; 5 Stembio Ltd., Entrepreneur 306, Asan-si 31538, Korea; yoon.st@yahoo.com (S.Y.); hsy4914@naver.com (S.-Y.H.); 6 Department of Medical Biotechnology, Soonchunhyang University, Asan-si 31538, Korea

**Keywords:** melatonin, extracellular vesicle-mimetic nanovesicles, transdermal delivery, atopic dermatitis, anti-inflammation

## Abstract

Atopic dermatitis (AD) is caused by multiple factors that trigger chronic skin inflammation, including a defective skin barrier, immune cell activation, and microbial exposure. Although melatonin has an excellent biosafety profile and a potential to treat AD, there is limited clinical evidence from controlled trials that support the use of melatonin as an AD treatment. The delivery of melatonin via the transdermal delivery system is also a challenge in designing melatonin-based AD treatments. In this study, we generated melatonin-loaded extracellular vesicle-mimetic nanoparticles (^Mela^NVs) to improve the transdermal delivery of melatonin and to evaluate their therapeutic potential in AD. The ^Mela^NVs were spherical nanoparticles with an average size of 100 nm, which is the optimal size for the transdermal delivery of drugs. ^Mela^NVs showed anti-inflammatory effects by suppressing the release of TNF-α and β-hexosaminidase in LPS-treated RAW264.7 cells and compound 48/80-treated RBL-2H3 cells, respectively. ^Mela^NVs showed a superior suppressive effect compared to an equivalent concentration of free melatonin. Treating a 2,4-dinitrofluorobenzene (DNCB)-induced AD-like mouse model with ^Mela^NVs improved AD by suppressing local inflammation, mast cell infiltration, and fibrosis. In addition, ^Mela^NVs effectively suppressed serum IgE levels and regulated serum IFN-γ and IL-4 levels. Taken together, these results suggest that ^Mela^NVs are novel and efficient transdermal delivery systems of melatonin and that ^Mela^NVs can be used as a treatment to improve AD.

## 1. Introduction

Atopic dermatitis (AD) is a skin disease characterized by a chronic inflammation of the skin. AD usually occurs in early childhood, and symptoms can persist even in adulthood. The disease burden of AD is increasing worldwide, with approximately 20% of children suffering from it [1,2]. AD causes pruritus, dryness, and other skin lesions, including serious exudate, excoriation, patches, and lichenification [3]. The symptoms may disappear with age; however, most children are also affected by other atopic diseases, such as allergic rhinitis and asthma [4,5,6]. The pathogenesis of AD involves the dysfunction of the skin barrier and inflammation, which are influenced by various environmental, immunological, and genetic factors. A recent study found that genetic defects of filaggrin, an epidermal skin barrier protein, were correlated with the development of AD [7,8]. In addition, an allergen can stimulate dendritic cells in the skin, causing Th2 lymphocytes to release high concentrations of pro-inflammatory cytokines (IL-4, IL-5, and IL-13) to activate serine proteases, which can impair the function of the skin barrier [9]. Although various treatments, such as topical corticosteroids, antihistamines, and immunosuppressants, are used, AD is difficult to treat, and long-term treatments have been known to cause adverse effects [10]. Therefore, there is a need to find a treatment that has few adverse effects and an excellent therapeutic effect.

Melatonin (N-acetyl-5-methoxytryptamine) is released by the pineal gland, cerebellum, bone marrow, and skin as an endogenous hormone [11]. The synthesis and secretion of melatonin is regulated in a circadian manner; therefore, melatonin regulates the circadian clock and sleep [12,13]. Melatonin exhibits antioxidant, anti-inflammatory, and anti-cancer activities, in addition to several other crucial properties [14,15,16,17]. Accumulating evidence has shown that melatonin can be used as a treatment for AD. Topical treatment with melatonin significantly inhibits the development of AD-like skin lesions. Melatonin reduces serum IgE levels and inhibits IL-4 and IFN-γ secretion from activated CD4^+^ T cells in dinitrofluorobenzene (DNFB)-treated NC/Nga mice [18]. In addition, melatonin protects keratinocytes from UV-induced apoptosis [19] and enhances the function of skin fibroblasts [20], thereby enhancing the role of the skin barrier. Although melatonin could be used as a treatment for AD, clinical trials investigating the therapeutic potential of melatonin in human patients with AD are limited. Therefore, the development of an efficient transdermal drug delivery system for melatonin could be helpful for the development of AD treatment. 

Extracellular vesicles (EVs) are nanometer-sized proteolipids that are naturally secreted by most cells, including bacteria, archaea, and eukaryotic cells. EVs contain cell-derived proteins, phospholipids, and nucleic acids, and deliver these molecules to target cells and change cell phenotypes [21,22]. Recently, studies have been carried out using EVs as drug delivery systems [23,24,25]. EVs have several advantages over conventional drug delivery systems, showing high stability in blood, biocompatibility, and tissue permeability [26,27]. In addition, EVs can be easily engineered to display target molecules. Although EVs have proven to be promising drug delivery systems, there are several difficulties associated with their clinical use, including low production yields and tedious isolation procedures.

Recently, EV-mimetic nanovesicles (NVs) have been developed to overcome the challenges of EVs. EV-mimetic NVs have biophysical characteristics similar to those of natural EVs, and they can be easily isolated with high yields [28,29,30]. EV-mimetic NVs can be generated using various methods, such as extrusion, sonication, high pH treatment, and microfluidic fabrication [30,31]. They can be loaded with several types of therapeutic drugs [32]. For example, doxorubicin-loaded EV-mimetic NVs showed targeted delivery to tumor tissue, with a superior anti-cancer effect compared to free doxorubicin [28]. In addition, dexamethasone-loaded EV-mimetic NVs showed excellent anti-inflammatory effects in a bacterial outer membrane vesicle (OMV)-induced SIRS model [29]. However, studies on melatonin-loaded EV-mimetic NVs (^Mela^NVs) as a treatment for AD have yet to be conducted. In this study, we showed that ^Mela^NVs have anti-inflammatory effects in LPS-treated RAW264.7 cells and compound 48/80-treated RBL-2H3 cells. The topical administration of ^Mela^NVs improved AD symptoms, indicating the potential of ^Mela^NVs as a treatment for AD.

## 2. Materials and Methods

### 2.1. Cell Culture 

Both human embryonic kidney 293 (HEK293) and RAW264.7 cells were cultured in Dulbecco’s Modified Eagle Medium with high glucose medium (HyClone) supplemented with 10% (*v*/*v*) fetal bovine serum (FBS) (Thermo Fisher Scientific, Waltham, MA, USA) and 100 U/mL penicillin/streptomycin (Thermo Fisher Scientific) at 37 °C and 5% CO_2_ in a humidified incubator. HEK293 cells were purchased from the American Type Culture Collection (Manassas, VA, USA) and RAW264.7 cells were obtained from the Korean Cell Line Bank (Seoul, Korea). 

### 2.2. Generation of Melatonin-Loaded Extracellular Vesicle-Mimetic NVs

HEK293 cells were scraped and resuspended at a concentration of 1 × 10^7^ cells/mL in PBS, with or without 1000 μg/mL of melatonin. Mini-extruders (Avanti Polar Lipids) were used for extruding cell suspensions, with a three-step extrusion process through 10-, 5-, and 1-μm polycarbonate membrane filters (Whatman). The filtrates underwent density gradient ultracentrifugation with iodixanol at 100,000× *g* for 1 h. Control NVs and ^Mela^NVs were concentrated between 10% and 50% iodixanol layers, and then extracted from the interface of the two layers for further experiments. The quantification of the total protein concentration in NVs and ^Mela^NVs was performed using a commercially available Bradford assay (Bio-Rad, Hercules, CA, USA).

### 2.3. Cryo-Electron Microscopy and Nanoparticle Tracking Analysis

For cryo-electron microscopy, 5 μL of NVs and ^Mela^NVs were placed onto 300-mesh EM carbon grids and frozen with liquid nitrogen using Vitrobot (Thermo Fisher Scientific). The sample was analyzed with Talos L120C cryo-TEM (Thermo Fisher Scientific) at a magnification of ×13,000 to obtain image data. The concentration of the particles of interest present in either NVs or ^Mela^NVs was measured using a NanoSight LM10 system (Malvern Instruments Ltd., Malvern, UK) and Nanoparticle Tracking Analysis software (version 2.3).

### 2.4. Western Blotting

Protein extracts from HEK293 cells, NVs, and ^Mela^NVs were separate by SDS-PAGE and transferred to poly(vinylidene fluoride) (PVDF) membranes. Subsequently, the membranes were blocked with 3% skim med milk for 1 h, followed by treatment with primary antibodies against CD81, CD9, and GM130. After washing the membrane three times with TBST (tris-buffered saline with 0.05% Tween-20), treatment with secondary antibodies was performed. After another round of washing with TBST, signals were detected using chemiluminescence (Amersham Pharmacia Biotech, Buckinghamshire, UK).

For the detection of COX-2, TNF-α, and protease-activated receptor-2 (PAR-2), mouse dorsal tissue was crushed with radioimmunoprecipitation (RIPA) buffer in the presence of protease inhibitors and phenylmethylsulfonyl fluoride. Then, proteins (25 μg per sample) were separated by SDS-PAGE and transferred onto PVDF membranes. The PVDF membranes were blocked with 5% bovine serum albumin solution at room temperature for 2 h and incubated at 4 °C for 18 h with the appropriate primary antibodies. The results were visualized using the Supernova ECL western blotting detection system (Cyanagen srl, Italy). Images were taken with Seni-Q 2000 (Lugen Sci Co., Ltd., Seoul, Republic of Korea) and the signal intensity was quantified using ImageJ software. The following commercially available antibodies were used: anti-PAR-2 (SC-13504) antibody, anti-CD81 (SC-166029) antibody, anti-β-Actin antibody (SC-47778), goat anti-rabbit IgG, and goat anti-mouse IgG conjugated to HRP purchased from Santa Cruz Biotechnology (Santa Cruz, CA, USA), and mouse anti-CD9 (555370) and mouse anti-GM130 (610822) antibodies purchased from BD Biosciences (San Diego, CA, USA). Anti-Cox2 (#122825) antibody and anti-TNF-α (#11948) antibody were obtained from Cell Signaling Technology (Danvers, MA, USA).

### 2.5. Quantification of Melatonin in Extracellular Vesicle-Mimetic NVs

Melatonin was quantified using a commercially available melatonin ELISA kit (Abcam, Cambridge, UK). Melatonin was extracted from the ^Mela^NVs using the ethyl acetate extraction method before performing ELISA. Briefly, 50 μg of ^Mela^NVs in protein amount was mixed with the same volume of cold ethyl acetate, and the sample was incubated on ice for 2 min. After centrifugation at 1000× *g* for 10 min at 4 °C, the upper organic layer containing melatonin was obtained. The sample was dried overnight under a stream of inert gas. The resulting pellet was reconstituted with a stabilizer solution provided with the kit, and the ELISA assay was conducted according to the manufacturer’s instructions. 

### 2.6. In Vitro β-Hexosaminidase Release Assay

RBL-2H3 cells were treated with 1 mg/mL compound 48/80 with or without NVs, ^Mela^NVs, or free melatonin. After incubation at 37 °C for 20 min, 50 μL of the supernatant was mixed with 200 μL of 1 mM p-nitrophenyl N-acetyl-beta-D-glucosamine in 50 mM citrate buffer (pH 4.5). The mixture was incubated again at 37 °C for 1 h. The reaction was quenched with 500 μL of sodium carbonate buffer (50 mM, pH 10.0). A microplate reader (BMG Labtech, Ortenberg, Germany) was used to measure the absorbance at 405 nm [33].

### 2.7. Animal Care

Eight-week-old male BALB/c mice (ORIENT Inc., Seongnam, Korea) were kept in cages under 25 °C and 12 h light/dark cycle conditions. Experiments involving animals were approved by the Institutional Animal Care and Use Committee of Soonchunhyang University (SCH20-0070).

### 2.8. Atopic Dermatitis-Like Mouse Model

The dorsal hair of five mice from each group was removed using hair removal cream. Mice were assigned to one of seven groups (five mice each): control (non-treated); 2,4-dinitrofluorobenzene (DNCB, negative control); dexamethasone (DEX, positive control; 2.5 mg/kg/day of DEX in distilled water); ^Mela^NVs (1000 μg/mL in protein concentrations); NVs without melatonin (same concentrate as ^Mela^NVs). Allergic inflammation was induced with 200 μL of 1% DNCB in 4:1 (*v*/*v*) acetone/olive oil administered topically on nine occasions [34]. Five days after the initial administration, 0.5% DNCB was applied to the backs and ears of the mice once every 16 days. After 45 days, all experimental and control groups of mice were sacrificed, and blood, ear, and dorsal skin samples were collected. 

### 2.9. Enzyme-Linked Immunosorbent Assay (ELISA)

To measure the serum IgE levels, mouse sera were obtained by centrifuging whole blood samples (20,000× *g* for 30 min at 4 °C). The resulting supernatant was collected for further experiments. For the determination of the IFN-γ levels, the mouse dorsal skins were sonicated three times each on an amplitude value (AMP) of 70% using a sonicator in RIPA buffer with 10% protease inhibitor cocktail (Millipore Cop., Billerica, MA, USA) and 10% phenylmethylsulfonyl fluoride (PMSF). The level of IFN-γ was quantified and normalized using an ELISA kit (BD Biosciences, San Diego, CA, USA). The ELISA plates were read at 450 nm on a Microplate reader (TECAN, Männedorf, Switzerland), and protein assay kit. The total protein levels were calculated as picograms per milligram (pg/mg). 

### 2.10. Histological Analysis

The dorsal skin samples were fixed with 10% formalin, dehydrated, and embedded in paraffin. For histological analysis, the samples were sectioned (5 μm) using an EXAKT grinding system (EXAKT 400CS; Norderstedt, Germany). Hematoxylin and eosin (H&E) staining was performed to measure dermal and epidermal thickness, Toluidine blue staining for detecting mast cell infiltration, and Masson trichrome staining to visualize collagen in the skin barrier. Slide scan images were obtained using APERIO CS2 and Image Scope software (Leica Microsystems, Buffalo Grove, IL, USA) was used for the calculations.

### 2.11. Statistical Analysis

All data are presented as the mean ± standard error of the mean (SEM). Statistical significance was determined by one- or two-way analysis of variance (ANOVA) between the groups. Tukey’s post-hoc test was used for three or more groups. The cutoff for statistical significance was *p* < 0.05.

## 3. Results

### 3.1. Generation and Characterization of NVs and Melatonin-Loaded NVs

**Figure 1:**
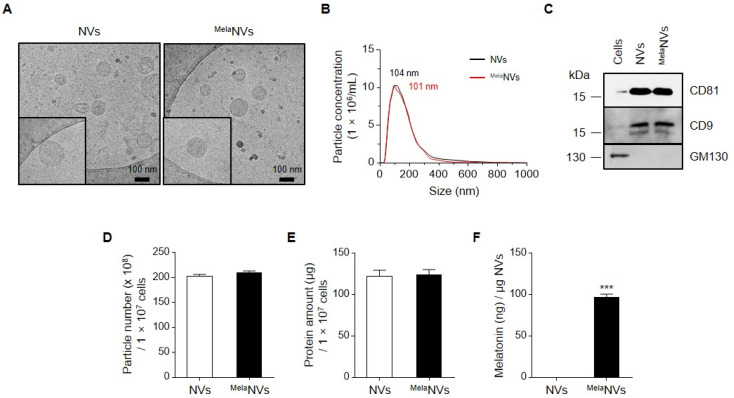
Comparison between extracellular vesicle-mimetic nanovesicles (NVs) and melatonin-loaded extracellular vesicle-mimetic nanovesicles (^Mela^NVs). (**A**) Representative images from cryo-electron microscopy data of NVs and ^Mela^NVs. Scale bar, 100 nm. (**B**) Distribution of the observed size of NVs and ^Mela^NVs. Nanoparticle tracking analysis were used for the measurement (*n* = 5). (**C**) Western blotting against CD81, CD9, and GM130 for cells, NVs, and ^Mela^NVs (10 μg of proteins). (**D**,**E**) Production yield of NVs and ^Mela^NVs in terms of particle number (**D**) and total protein (**E**) when 1 × 10^7^ HEK293 cells were used (*n* = 5). (**F**) Quantification of melatonin in NVs and ^Mela^NVs using melatonin ELISA kit (*n* = 3). Data are presented as mean ± SEM. *** *p* < 0.001 vs. NVs group.

To produce control NVs and ^Mela^NVs, HEK293 cells were harvested and resuspended at a concentration of 1 × 10^7^ cells/mL in PBS with or without 500 μg/mL melatonin. The cell suspension was extruded using 10-, 5-, and 1-μm pore-sized polycarbonate membranes. The NVs and ^Mela^NVs were purified from the interface of 10% and 50% iodixanol after density gradient ultracentrifugation. Cryo-electron microscopy images showed that NVs and ^Mela^NVs had a spherical nanovesicular morphology without noticeable damage (Figure 1A). The nanoparticle tracking analysis results showed that the NVs and ^Mela^NVs had a mode size of approximately 100 nm, with a size distribution range of 30–500 nm (Figure 1B). The western blotting results showed that NVs and ^Mela^NVs were enriched with EV marker proteins, such as CD81 and CD9, and de-enriched with GM130, which is a Golgi marker protein (Figure 1C). From 1.0 × 10^7^ of HEK293 cells, 2.03 × 10^10^ and 2.10 × 10^10^ particles of NVs and ^Mela^NVs were obtained, respectively (Figure 1D). Moreover, from 1.0 × 10^7^ of HEK293 cells, 122.4 and 124.2 μg of NVs and ^Mela^NVs were obtained, respectively (Figure 1E). The ratio of particles to μg protein of NVs and ^Mela^NVs were 1.57 × 10^10^ and 1.62 × 10^10^ particles/μg, respectively. The melatonin loading amount in ^Mela^NVs was determined using a melatonin ELISA kit. The results showed that 1 μg of ^Mela^NVs contained 97.1 ng of melatonin (Figure 1F). These results indicate that ^Mela^NVs are EV-mimetic NVs containing melatonin, suggesting that ^Mela^NVs can be used for the efficient delivery of melatonin.

### 3.2. In Vitro Anti-Inflammatory Effect of ^Mela^NVs in RAW264.7 and RBL-2H3 Cells

**Figure 2:**
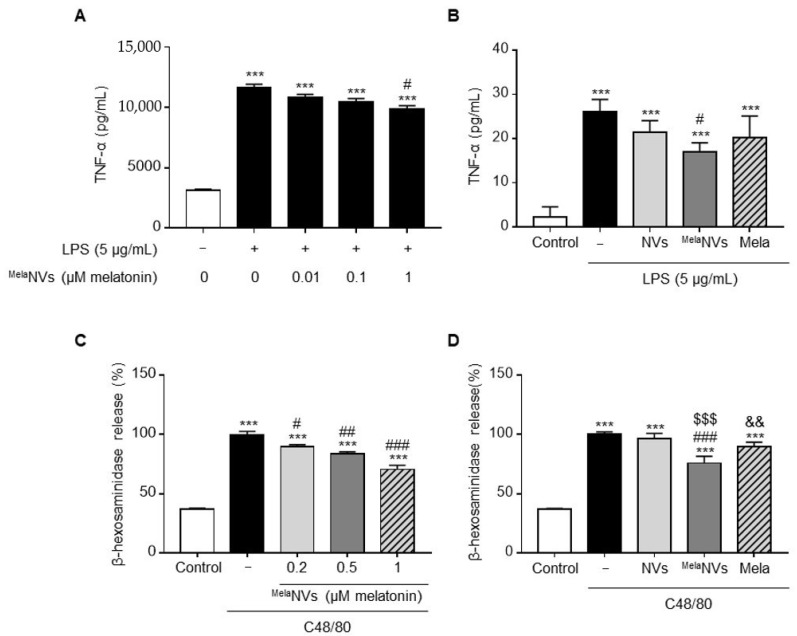
In vitro anti-inflammatory effect of ^Mela^NVs. (**A**) Quantification of TNF-α in conditioned medium of ^Mela^NVs-treated RAW264.7 cells under the LPS condition (*n* = 3). *** *p* < 0.001 versus control. (**B**) Comparison of anti-inflammatory effects of ^Mela^NVs and free melatonin in RAW264.7 cells under the LPS condition (*n* = 3). (**C**,**D**) Quantification of β-hexosaminidase in conditioned medium of RBL-2H3 cells either treated with NVs, ^Mela^NVs, or free melatonin under C48/80 treatment condition (*n* = 3). Data are presented as mean ± SEM. *** *p* < 0.001 vs. control, # *p* < 0.05, ## *p* < 0.01, ### *p* < 0.001 vs. C48/80 group, $$$ *p* < 0.001 vs. NVs group, && *p* < 0.01 vs. ^Mela^NVs group. LPS: lipopolysaccharides; C48/80: compound 48/80; Mela: melatonin.

To determine the anti-inflammatory effect of ^Mela^NVs, whether ^Mela^NVs could suppress the inflammatory response of LPS-treated RAW264.7 cells was tested. The treatment of RAW264.7 cells with 5 μg/mL of LPS induced the release of the pro-inflammatory cytokine TNF-α. Co-treatment of ^Mela^NVs with LPS resulted in the suppression of TNF-α release in a dose-dependent manner. ^Mela^NVs treated with 1 μM melatonin inhibited TNF-α release by 15% compared to the LPS-treated group (Figure 2A). Treatment with higher doses of ^Mela^NVs did not increase the suppression of TNF-α release (data not shown). EV-mimetic formulations of drugs are known to enhance the drug delivery efficacy of loaded drugs, possibly via interactions with membrane proteins between the cells and vesicles [28,29]. To determine whether ^Mela^NVs exhibited superior anti-inflammatory effects compared to the same amount of free melatonin, RAW264.7 cells were treated with ^Mela^NVs or the same amount of free melatonin, and their anti-inflammatory effects were compared. The results showed that ^Mela^NVs had better anti-inflammatory effects compared to the same amount of free melatonin (Figure 2B). We further confirmed the anti-inflammatory effects of ^Mela^NVs in C48/80-stimulated RBL-2H3 cells. RBL-2H3 cells, which are basophil cell lines, are functionally homologous to mucosal mast cells and are mainly used to study allergic reactions. The treatment of RBL-2H3 cells with 1 mg/mL of compound 48/80 (C48/80) induced the release of β-hexosaminidase, which is frequently used to measure the degree of mast cell degranulation. Co-treatment of ^Mela^NVs with C48/80 significantly inhibited β-hexosaminidase release in a dose-dependent manner (Figure 2C). Co-treatment of RBL-2H3 with C48/80 and NVs without melatonin did not induce changes in β-hexosaminidase release. However, co-treatment with C48/80 and ^Mela^NVs (in 1 μM melatonin) inhibited β-hexosaminidase release by 25%, while 1 μM of free melatonin inhibited β-hexosaminidase release by 10% (Figure 2D). These results suggest that ^Mela^NVs can maximize the anti-inflammatory effect of melatonin by facilitating the delivery of melatonin into cells.

### 3.3. ^Mela^NVs Ameliorate DNCB-Induced AD and Suppress Mast Cell Infiltration and Fibrosis in AD-like Skin Lesions

**Figure 3:**
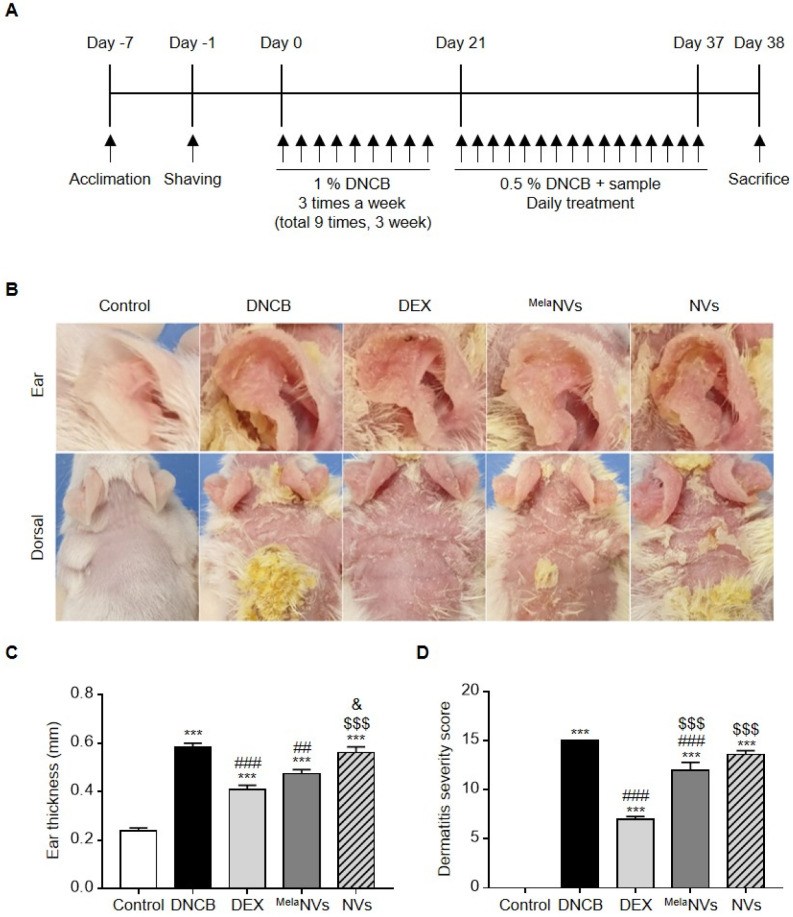
^Mela^NVs improve AD symptoms and reduce the dermatitis severity score in the DNCB-induced AD mouse model. (**A**) Schematic diagram of experimental schedule. AD-like skin lesions were induced in ears and dorsal skin of BALB/c mice with 200 μL of 1% DNCB (administered 9 times). Mice were treated with 0.5% DNCB in the presence DEX (2.5 mg/kg), NVs (1000 μg/mL protein), or ^Mela^NVs (1000 μg/mL protein, 1 mg/kg in melatonin) daily for 16 days. (**B**) Representative images of ear and dorsal skin of mice in each group on day 37. (**C**) Measure of ear thickness on day 37 (*n* = 5). (**D**) Dermatitis scores (SCORAD) on day 37 (*n* = 5). Data are presented as mean ± SEM. *** *p* < 0.001 vs. control, ## *p* < 0.01, ### *p* < 0.001 vs. DNCB group, $$$ *p* < 0.001 vs. DEX group, & *p* < 0.05 vs. ^Mela^NVs group.

We investigated whether ^Mela^NVs could improve the symptoms of AD in a DNCB-induced AD mouse model. We established an AD mouse model by treating the ears and dorsal skin of mice with 1% DNCB three times a week for 3 weeks (9 times in total). The ears and dorsal skin were treated with ^Mela^NVs in the presence of 0.5% DNCB once a day for 16 days (Figure 3A). Treatment with DNCB induced AD-like skin lesions showing erythema, edema, and dryness, suggesting that DNCB successfully induced AD-like phenotypes (Figure 3B,C). The topical administration of ^Mela^NVs significantly improved the symptoms of AD by reducing skin inflammation and ear thickness. However, treatment with NVs did not improve the AD-like phenotypes (Figure 3B,C). We also measured dermatitis severity scores according to previously described criteria [35]. The administration of ^Mela^NVs significantly reduced dermatitis severity scores compared to DNCB- and NV-treated mice (Figure 3D).

**Figure 4:**
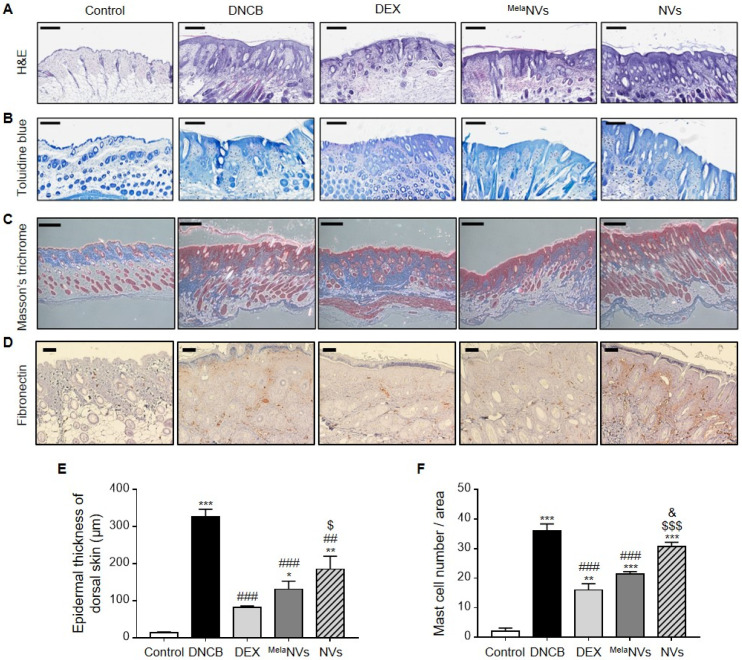
^Mela^NVs treatment decreases epidermal thickness and suppresses mast cell infiltration and fibrosis in AD-like skin lesions. (**A**–**D**) Representative images of hematoxylin and eosin (H&E) staining (scale bar, 300 μm) (**A**), toluidine blue staining (scale bar, 200 μm) (**B**), Masson’s trichrome Scheme 100 μm) (**C**), and immunohistochemical staining (scale bar, 100 μm) (**D**) of dorsal skin of mice in each group. (**E**) Comparison of epidermal thickness of dorsal skin lesions for different experimental groups determined by H&E staining. (**F**) Observed infiltration of mast cells in dorsal skin lesions determined by toluidine blue staining. Data are presented as mean ± SEM. * *p* < 0.05, ** *p* < 0.01, *** *p* < 0.001 vs. control, ## *p* < 0.01, ### *p* < 0.001 vs. DNCB group, $ *p* < 0.01, $$$ *p* < 0.001 vs. DEX group, & *p* < 0.05 vs. ^Mela^NVs group.

H&E staining of dorsal skin showed that the application of ^Mela^NVs effectively reduced the epidermal thickness of the dorsal skin (Figure 4A). The measurement of the epidermal thickness further strengthened H&E results (Figure 4E).

Next, we assessed mast cell infiltration in AD-like skin lesions by performing toluidine blue staining of the dorsal skin of mice in each group. As a result, DNCB treatment was found to considerably increase the number of mast cells in the AD-like skin lesions. Treatment with ^Mela^NVs effectively suppressed the infiltration of mast cells into the dorsal skin, while the NV treatment did not show a significant decrease in mast cell infiltration (Figure 4B,F). Next, we evaluated the protective effects of ^Mela^NVs against fibrosis in AD-like skin lesions. Fibrotic remodeling is a major characteristic of AD-like skin lesions. Collagen and fibronectin deposition in the dorsal skin were determined using Masson’s trichrome staining and immunohistochemistry, respectively. Masson’s trichrome staining of dorsal skin showed that collagen deposition was effectively reduced in ^Mela^NVs-treated mice compared to that in DNCB-treated mice (Figure 4C). The immunohistochemistry results also showed that fibronectin expression in AD-like skin lesions was reduced in ^Mela^NV-treated mice (Figure 4D). These results suggest that ^Mela^NVs effectively improved AD symptoms and inhibited the infiltration of mast cells and fibrosis in AD-like skin lesions.

### 3.4. ^Mela^NVs Decrease Serum IgE Levels, Restore Th1/Th2 Cytokine Balance, and Suppress the Release of Inflammatory Cytokines in AD-like Skin Lesions

**Figure 5:**
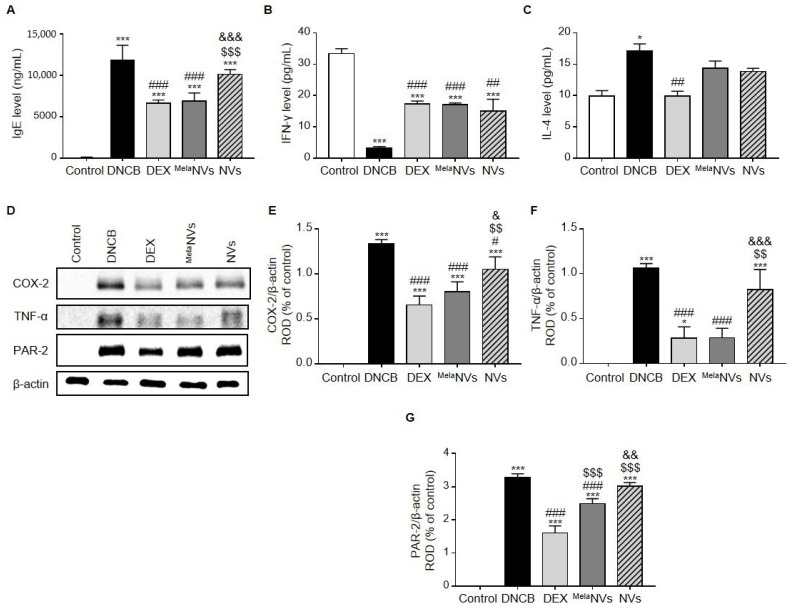
Effect of ^Mela^NVs on serum IgE, IFN-γ, and IL-4 levels and on the expression of pro-inflammatory cytokines in AD-like skin lesions. (**A**) Serum IgE levels after treatment of DEX or ^Mela^NVs or NVs in DNCB-induced AD mice (*n* = 5). (**B**,**C**) Serum levels of IFN-γ (**B**) and IL-4 (**C**) determined by using ELISA (*n* = 5). (**D**–**G**) Western blot analysis of COX-2, TNF-α, and PAR-2 in dorsal AD-like skin lesions in the respective experimental groups. Protein expression was measured relative to β-actin with densitometry. Data are presented as mean ± SEM. * *p* < 0.05, *** *p* < 0.001 vs. control, # *p* < 0.05, ## *p* < 0.01, ### *p* < 0.001 vs. DNCB group, $$ *p* < 0.01, $$$ *p* < 0.001 vs. DEX group, & *p* < 0.05, && *p* < 0.01, &&& *p* < 0.001 vs. ^Mela^NVs group.

To determine whether the topical administration of ^Mela^NVs could reduce serum IgE levels and restore Th1/Th2 cytokine balance, serum IgE, IFN-γ, and IL-4 levels were evaluated by ELISA. As a result, DNCB treatment was found to significantly increase the serum IgE levels compared to the controls (Figure 5A). In addition, the topical application of ^Mela^NVs significantly reduced serum IgE levels, whereas treatment with NVs did not cause a significant reduction in serum IgE levels (Figure 5A). DNCB treatment lowered serum IFN-γ levels and increased the serum IL-4 levels. Treatment with ^Mela^NVs significantly increased IFN-γ levels and decreased IL-4 levels, although this was not statistically significant (Figure 5B,C). Interestingly, treatment with NVs also significantly increased the serum IFN-γ levels (Figure 5B). Despite a decrease in the IL-4 levels after NV treatment, no significant difference was observed compared to the DNCB group (Figure 5C). 

Then, whether ^Mela^NVs could suppress the pro-inflammatory cytokines in AD-like skin lesions was investigated. As a result, the expression of COX-2 and TNF-α, both of which are known to promote inflammation, was found to be suppressed in AD-like skin lesions (Figure 5D–F). Protease-activated receptor-2 (PAR-2) is known to be activated in AD and is associated with the release of pro-inflammatory cytokines and the disruption of the skin barrier in AD-like skin lesions [36]. The topical administration of ^Mela^NVs effectively suppressed PAR-2 expression in AD-like skin lesions (Figure 5D,G) compared to the DNCB- and NV-treated groups. These results suggest that ^Mela^NVs decreased serum IgE levels, balanced Th1/Th2 cytokine levels, and suppressed the expression of pro-inflammatory cytokines.

## 4. Discussion

Although the precise pathogenesis of AD has yet to be fully elucidated, a defective skin barrier, immune cell activation (mast cells and Th1 and Th2 cells), and environmental factors are known to be involved in the progression of AD [37]. Melatonin is a powerful antioxidant that can help to maintain skin barrier function by protecting keratinocytes from apoptosis [19] and enhancing the function of skin fibroblasts [20]. In addition, the peritoneal injection of melatonin to the DNFB-treated AD mouse model inhibits serum IgE levels and cytokine secretion of IL-4 and IFN-γ from activated CD4^+^ T cells [18]. Melatonin has also been shown to inhibit the degranulation, infiltration, and activation of mast cells [38]. In our previous studies, we demonstrated that melatonin suppresses high glucose- or *p*-Cresol-induced fibrosis in human renal proximal tubule epithelial cells [39,40]. Based on these results, we hypothesized that melatonin-loaded EV mimetics would have excellent inhibitory effects on AD. In this study, we demonstrated that ^Mela^NVs improved the symptoms of AD and inhibited mast cell infiltration and fibrosis in AD-like skin lesions. In addition, we showed that ^Mela^NV decreased IgE levels, restored IFN-γ and IL-4 levels, and inhibited the inflammatory response in AD-like skin lesions. While Kim et al. intraperitoneally injected 20 mg/kg of melatonin to observe its inhibitory effects on AD, in this study, we topically administered 1 mg/kg of ^Mela^NVs. This suggests that the transdermal delivery of melatonin using ^Mela^NVs can increase treatment efficacy and reduce the potential adverse effects of melatonin.

Several types of delivery systems, including ethyl cellulose nanoparticles [41], lipid carriers [42], and polycaprolactone nanoparticles [43], have been developed to increase the efficiency of melatonin delivery. Bessonea et al. demonstrated that ethyl cellulose nanocapsules loaded with melatonin have a superior melatonin delivery efficiency to the cornea compared to free melatonin [41]. In our study, we demonstrated that ^Mela^NVs have better anti-inflammatory effects than the same amount of melatonin in C48/80-stimulated RBL-2H3 cells (Figure 2C). Compared with other drug delivery systems, EV-mimetics have several advantages, including ease of displaying targeting molecules on the surface NVs when the cells are genetically engineered to express the targeting molecules and have low immunogenicity, especially when patient-derived cells are used for NV generation. Although immune reactions were not observed in the NV- or ^Mela^NV-treated groups, the potential toxicity of EV-mimetics should be further verified in future studies. 

In the in vivo experiment, treatment with bare NVs also showed protective effects, particularly in terms of epidermal thickness and the IFN-γ, COX2, and TNF-α levels. Although NVs have been used as a melatonin drug delivery system, NVs may have anti-inflammatory functions. Tao et al. generated lncRNA-H19 containing EV-mimetic nanovesicles (^H19^EMNVs) by extruding H19-overexpressing HEK293 cells. ^H19^EMNVs treatment in a diabetic rat model showed increased healing processes in chronic wounds [44]. Interestingly, control EMNV were also found to exert effects on in vitro tube formation, in vivo wound healing, and angiogenesis [44]. Park et al. generated EV-mimetics from human bone marrow-derived MSCs and demonstrated the anti-inflammatory effect of MSC-derived NVs in bacterial OMV-treated RAW264.7, and in an OMV-induced sepsis model [45]. Proteins related to angiogenesis, inflammation, and stress protection, such as Jaggec-1 ligand protein, CD73, and 14-3-3 proteins, were found to exist in NVs. Likewise, certain proteins or RNAs present in HEK293-derived NVs may exert their anti-atopic dermatitis effects in a mouse model.

In our study, ^Mela^NVs reduced DNCB-induced PAR-2 expression in AD-like skin lesions (Figure 5G,H). PAR-2 has been associated with epidermal barrier homeostasis, immune response, pigmentation, fibrosis, and pruritus in the skin [46,47,48,49,50], and is known to play a role in the pathogenesis of AD [51,52]. The PAR-2 agonist increased itch and induced the irresponsiveness of sensory nerves to anti-histamine [53]. Furthermore, PAR-2 affects epidermal keratinocytes to generate the AD-aggravating factor, thymic stromal lymphopoietin (TSLP) [54]. In Netherton syndrome, which is another skin disease that has been associated with AD, a loss-of-function mutation in the serine protease inhibitor of kazal type 5) gene, which encodes LEKTI (lympho-epithelial kazal type related inhibitor type 5 (*SPINK5*) in humans, was reported to play a significant role. Here, LEKTI has been found to primarily regulate tissue kallikrein 5 (KLK5) and kallikrein 7 (KLK7) [55]. In keratinocytes, KLK7 overexpression results in AD [56], and excessive KLK5 activates PAR-2 [57]. Therefore, the loss of *SPINK5* gene functions upregulates KLK5, which in turn enhances the expression of TSLP, which leads to impairment of the junctional integrity of the skin during development [58].

In conclusion, our data provide experimental evidence for:

- The anti-inflammatory effects of ^Mela^NVs in LPS-stimulated RAW264.7 cells and C48/80-stimulated RBL-2H3 cells.
- We demonstrated that ^Mela^NVs could improve AD-like symptoms and suppress mast cell infiltration and fibrosis in AD-like skin lesions. ^Mela^NVs decreased serum IgE, restored IFN-γ and IL-4 levels, and inhibited COX-2, TNF-α, and PAR-2 expression.
- These results suggest that ^Mela^NVs have an excellent anti-atopic effect and can be used for the treatment of AD.
- As ^Mela^NVs are an excellent melatonin delivery system, ^Mela^NVs may also be used in other skin disease models, including decubitus and eye disease, to deliver melatonin.
